# *Streptococcus mutans* regulates ubiquitin modification of *Candida albicans* in the bacterial-fungal interaction

**DOI:** 10.1371/journal.ppat.1012887

**Published:** 2025-02-03

**Authors:** Yixin Zhang, Zhen Gu, Zhengyi Li, Qinrui Wu, Xin Xu, Xian Peng

**Affiliations:** 1 State Key Laboratory of Oral Diseases & National Center for Stomatology & National Clinical Research Center for Oral Diseases, Chengdu, China; 2 Department of Cariology and Endodontics, West China School of Stomatology, Sichuan University, Chengdu, China; Chinese Academy of Sciences, CHINA

## Abstract

The ecological interplay between *Streptococcus mutans* and *Candida albicans* within dental plaque biofilms is an important factor driving pathogenesis of dental caries. This study aimed to investigate *S*. *mutans* regulation of *C*. *albicans* growth and virulence through extracellular membrane vesicles (EMVs) and modulation of ubiquitination, a key protein post-translational modification. We established a transwell co-culture model to enable “contact-independent” interactions between *S*. *mutans* and *C*. *albicans*. *S*. *mutans* EMVs were found to directly associate with *C*. *albicans* cells and promote biofilm formation and growth. Quantitative ubiquitination profiling revealed *S*. *mutans* dramatically alters the ubiquitination landscape in *C*. *albicans*. We identified 10,661 ubiquitination sites across the *C*. *albicans* proteome and their enrichment in pathways related to translation, metabolism, and stress adaptation. Co-culture with *S*. *mutans* led to upregulation of ubiquitination on 398 proteins involved in sugar catabolism and generation of reducing power. *S*. *mutans* upregulated ubiquitination of superoxide dismutase-3 of *C*. *albicans*, inducing its degradation and heightened reactive oxygen species levels, and concomitantly stimulated *C*. *albicans* growth. Our findings elucidate EMVs and ubiquitination modulation as key mechanisms governing the *S*. *mutans-C*. *albicans* interplay and provide new insights into the promotion of a cariogenic oral biofilm environment. This study significantly advances understanding of the complex molecular interactions underlying dental plaque dysbiosis and caries pathogenesis.

## Author summary

Dental caries result from an imbalance in the oral microbiome, with opportunistic pathogens like *Streptococcus mutans* and *Candida albicans* becoming dominant. Recent FISH imaging has revealed spatial segregation between *S*. *mutans* and *C*. *albicans* in dental plaque in situ, implying "contact-independent" interactions. In this study, we discovered that *S*. *mutans* can promote *C*. *albicans* growth and biofilm formation through secreted extracellular membrane vesicles (EMVs) that directly associate with *C*. *albicans* cells and contain bioactive cargos that modulate cell signaling. Through advanced quantitative profiling, we revealed that *S*. *mutans* dramatically alters ubiquitination, an important protein modification governing stability and activity, across the *C*. *albicans* proteome. Specifically, *S*. *mutans* increased ubiquitination of enzymes involved in sugar metabolism, potentially enhancing energy production. *S*. *mutans* also triggered ubiquitination and degradation of a superoxide dismutase enzyme in *C*. *albicans*, leading to elevated reactive oxygen species levels and paralleled by an increase in the growth of *C*. *albicans*. Overall, our findings provide novel insights into EMV-mediated communication and ubiquitination dynamics as key mechanisms by which *S*. *mutans* modulates *C*. *albicans* physiology and promotes a cariogenic microbiome environment. This significantly furthers our understanding of dental plaque microbial interplay and its contributions to oral disease pathogenesis.

## Introduction

*Streptococcus mutans*, a Gram-positive bacterium, is widely recognized as a major culprit in dental caries [1]. *Candida albicans*, frequently found in the oral cavity, is an opportunistic fungus closely linked to both early childhood caries (ECC) and root caries [2,3]. The interplay between *S*. *mutans* and *C*. *albicans* is crucial in altering the balance of dental plaque microbiota, thereby facilitating dental caries’ emergence and progression. Co-infections of *S*. *mutans* and *C*. *albicans* have been observed in plaque samples from ECC, significantly contributing to severe early childhood caries (S-ECC) [4–6]. *S*. *mutans* produces glucosyltransferases B (GtfB) that adheres to mannans on *C*. *albicans*’ surface, utilizing sucrose to synthesize extracellular polysaccharides (EPS) in situ. This interaction strengthens the mutual adherence of the bacteria and fungi, leading to the development of complex biofilms [7–9]. Additionally, *C*. *albicans* can incite the production of EPS and trigger the activation of various virulence factors in *S*. *mutans* [10]. The intricate interplay between bacteria and fungi exerts a significant influence on the cariogenicity regulation of dental plaque [11]. Recent studies examining microbial distribution in carious lesions found that within dental plaque, *C*. *albicans* often forms corncob structures with non-mutans streptococci at the biofilm surface layer, while *S*. *mutans* tends to form isolated colonies and does not usually come in close contact with *C*. *albicans* [12]. This indicates that a "contact-independent" interaction may play a crucial role in the dynamic between *S*. *mutans* and *C*. *albicans*.

Extracellular membrane vesicles (EMVs) have emerged as important mediators of intercellular communication and regulation of microbial interactions. EMVs contain diverse cargos of proteins, lipids, and nucleic acids that can modulate virulence, biofilm formation, and gene expression in recipient cells [13,14]. *S*. *mutans* extracellular membrane vesicles (*S*.*m* EMVs) play a key role in *S*. *mutans* biofilm formation and inter-species interactions [15,16]. *S*.*m* EMVs can promote *C*. *albicans* biofilm formation and carbohydrate metabolism [17]. However, the specific effects of *S*. *m* EMVs on *C*. *albicans* growth and pathogenesis remain to be fully elucidated.

Ubiquitin is a highly conserved small polypeptide composed of 76 amino acids, covalently linked to free amino groups on target proteins through its carboxyl-terminal glycine residue. During the process of ubiquitination, ubiquitin-activating enzyme E1, ubiquitin-conjugating enzyme E2, and ubiquitin ligase E3 cooperate to attach ubiquitin molecules to target proteins [18]. Ubiquitination is a crucial post-translational modification that regulates protein stability, function, and subcellular localization. The attachment of single or poly-ubiquitin chains to target proteins can signal proteasomal degradation or influence protein activity and molecular interactions [19,20]. Prior studies have uncovered critical roles for ubiquitination in regulating growth, stress adaptation, and virulence in *C*. *albicans* [21]. However, the ubiquitination landscape in *C*. *albicans* and its dynamics during interactions with oral microbes such as *S*. *mutans* have yet to be characterized.

This study aimed to investigate *S*. *mutans* regulation of *C*. *albicans* growth and biofilm formation via a “contact-independent” mechanism involving EMVs. We further employed quantitative ubiquitination profiling to elucidate the effects of *S*. *mutans* on the ubiquitination landscape in *C*. *albicans* and explore the underlying mechanisms governing their interplay. Our findings provide new insights into the functional interactions between these major dental plaque inhabitants and their implications for oral microbial ecology and dental caries pathogenesis.

## Results

### *S*.*m* EMVs promotes *C*. *albicans* growth and biofilm formation

**Fig 1:**
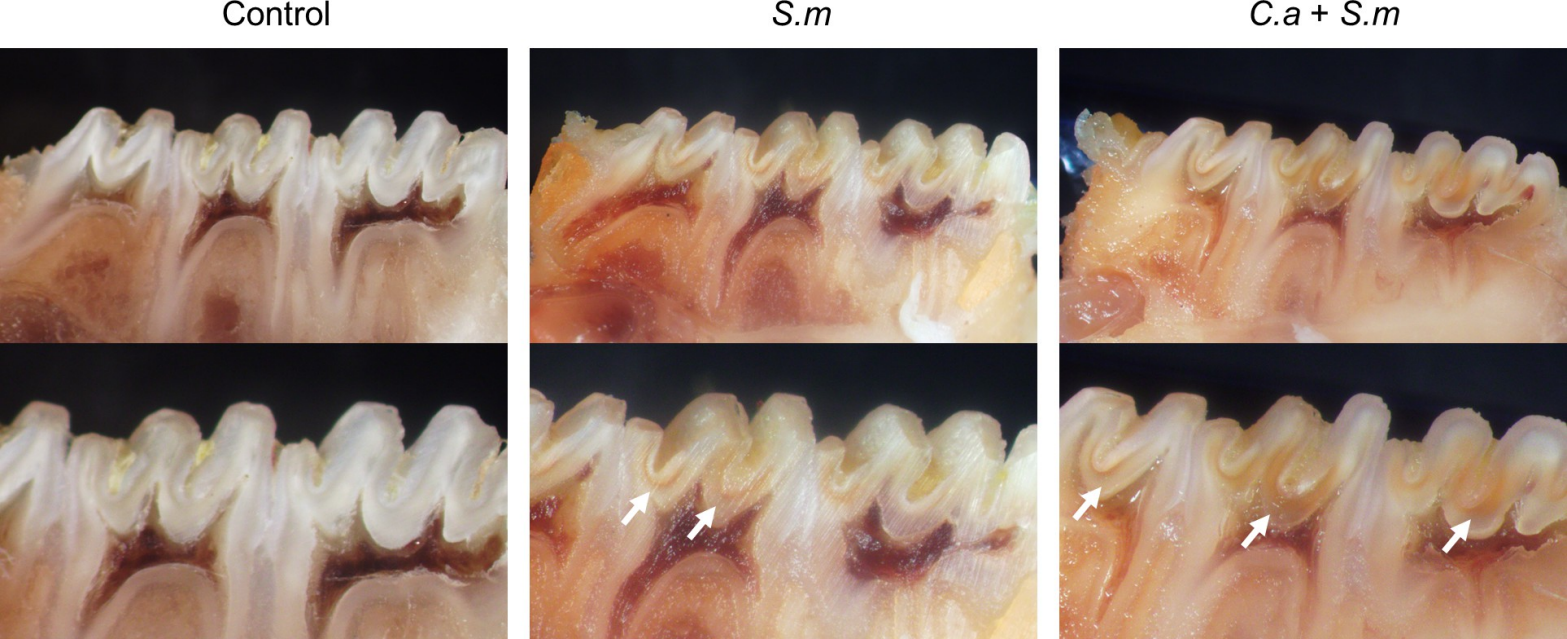
The co-infection of *S*. *mutans* and *C*. *albicans* promotes the process of caries. The sulcal view of the maxillaries of the model rats, with arrows indicating representative caries lesions.

Firstly, we evaluated the impact of the co-infection of *S*. *mutans* and *C*. *albicans* on the development of caries lesions in the rat model. The evaluation of caries by Keyes’ scoring method revealed that, compared with the control group and the group infected with *S*. *mutans*, the animals with co-infection presented more severe caries lesions on sulcal (Fig 1 and Table 1).

**Table 1:** Statistical chart of the caries scores.

Infecting	Mean Sulcal caries scores (n = 7)			
	E	Dm	Ds	Dx
Control	18.6 ± 1.40^a^	11.4 ± 2.07^a^	1.7 ± 0.95^a^	0.1 ± 0.38^a^
*S*.*m*	29.4 ± 1.90^b^	23.6 ± 1.71^b^	6.6 ± 0.98^b^	1.3 ± 0.95^b^
*S*.*m* + *C*.*a*	32.9 ± 1.86^c^	22.1 ± 1.68^b^	8.6 ± 0.98^c^	3.0 ± 1.15^c^

Groups with the same letter indicate no significant difference between them (p > 0.05), while groups with different letters indicate a significant difference between them (p < 0.05).

**Fig 2:**
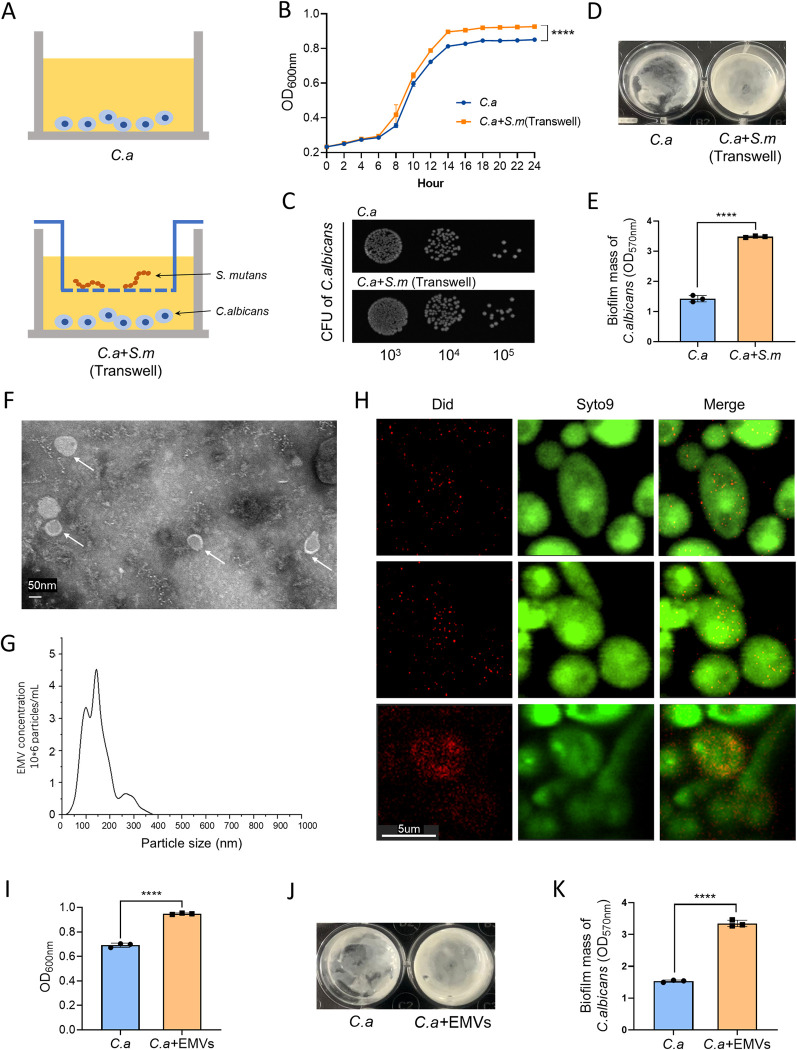
Interaction between *S*. *mutans* and *C*. *albicans* via EMVs. (A) Diagram of the transwell co-culture model depicting the spatial separation of *S*. *mutans* and *C*. *albicans*. (B) Influence of *S*. *mutans* on the Growth Kinetics of *C*. *albicans*. (C) Colony-Forming Units (CFU) of *C*. *albicans*. (D) Impact of *S*. *mutans* on *C*. *albicans* Biofilm Formation. (E) Biofilm biomass quantification of *C*. *albicans*. (F) TEM image of negative-stained *S*.*m* EMVs, highlighting spherical vesicles (indicated by white arrows). (G) NTA profile depicting the size distribution of *S*.*m* EMVs. (H) Fluorescent visualization of *S*.*m* EMVs (DiD, red) in co-culture with *C*. *albicans* cells (SYTO-9, green), indicating co-localization. (I) *S*.*m* EMVs’ effect on the planktonic growth of *C*. *albicans*. (J) Effect of *S*.*m* EMVs on biofilm formation of *C*. *albicans*. (K) Quantitative analysis of *C*. *albicans* biofilm biomass. *S*.*m*: *Streptococcus mutans*, *C*.*a*: *Candida albicans*, EMVs: *Streptococcus mutans* extracellular membrane vesicles, ****: P < 0.0001.

To investigate the mechanism by which co-infection promotes the occurrence of caries, in our investigation, we utilized a transwell setup to establish a "contact-independent" co-culture system, wherein *C*. *albicans* was positioned on the lower plate and *S*. *mutans* was placed in the upper compartment of the transwell insert. This arrangement allowed for shared nutrient access and metabolic product exchange without direct contact between the two species (Fig 2A). Relative to solo cultures, co-culturing with *S*. *mutans* resulted in augmented growth of *C*. *albicans*, evident from increased biomass and colony-forming units (Fig 2B and 2C), as well as a significant enhancement in *C*. *albicans* biofilm biomass (Fig 2D and 2E).

Our hypothesis posits that *S*. *mutans* promotes *C*. *albicans* growth and biofilm formation through the secretion of EMVs. To investigate this, EMVs were isolated from *S*. *mutans* culture media using ultracentrifugation and characterized. Transmission electron microscopy (TEM) imaging revealed spherical vesicular structures of the extracted *S*.*m* EMVs (Fig 2F). The average diameter of these EMVs was found to be 150.3 nm through nanoparticle tracking analysis (NTA) (Fig 2G). Following staining with DiD (red) for *S*.*m* EMVs and SYTO-9 (green) for *C*. *albicans* cells, confocal laser scanning microscopy (CLSM) allowed for the visualization of *S*.*m* EMVs co-localizing with *C*. *albicans* after an hour of incubation, indicating that *S*.*m* EMVs were taken up by *C*. *albicans* through endocytosis. (Fig 2H). This interaction led to a pronounced increase in both growth and biofilm formation of *C*. *albicans* in the presence of *S*.*m* EMVs, compared to control groups (Fig 2J and 2K). These results provide solid evidence for the role of *S*. *m* EMVs in the "contact-independent" facilitation of *C*. *albicans* proliferation.

### *S*.*m* EMVs influence protein ubiquitination in *C*. *albicans*

**Fig 3:**
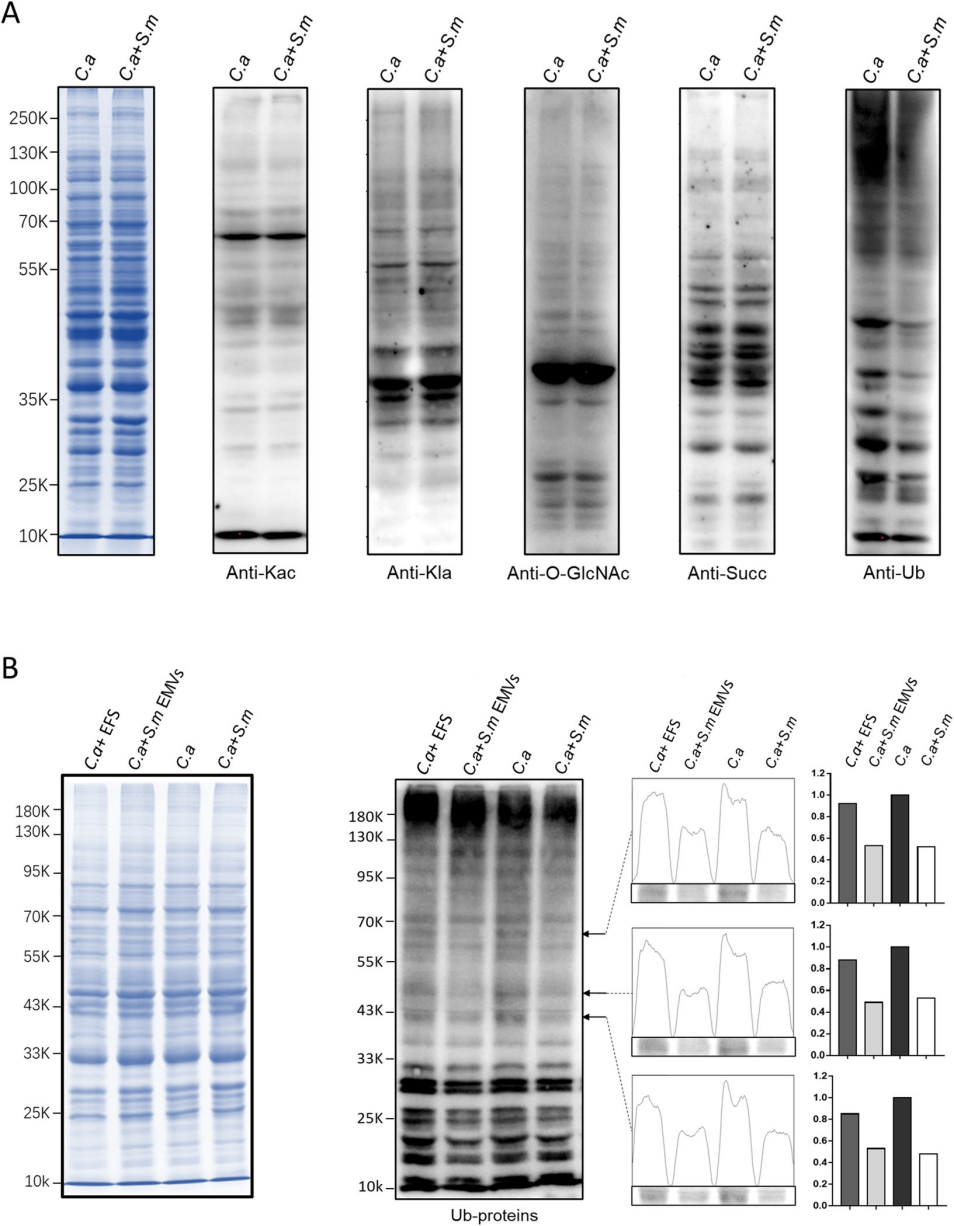
(A) Left: Coomassie Brilliant Blue staining of total *C*. *albicans* proteins; Right: Immunoblot analysis of various post-translational modifications in total *C*. *albicans* proteins, highlighting ubiquitination patterns. (B) Left: Total protein staining of *C*. *albicans*; Right: Immunoblot of ubiquitination modifications in *C*. *albicans* proteins treated with *S*.*m* EMVs. *S*.*m*: *Streptococcus mutans*, *C*.*a*: *Candida albicans*, EMV: *Streptococcus mutans* extracellular membrane vesicles. EFS: EMVs-free supernatants of *S*. *mutans* cultures.

We utilized post-translational modification-specific antibodies to examine changes in protein post-translational modifications of *C*. *albicans* when co-cultured with *S*. *mutans* via the transwell model. The co-culture group resulted in a significant reduction in the ubiquitination of *C*. *albicans* proteins, whereas other post-translational modifications like acetylation, lactylation, glycosylation, and succinylation remained non significantly changed (Fig 3A). These patterns, consistently replicated across three biological replicates, demonstrate *S*. *mutans*’ specific and consistent influence on the ubiquitin landscape of *C*. *albicans* proteins (S1 Fig). Further investigation with isolated *S*.*m* EMVs treated *C*. *albicans* showed distinct ubiquitination patterns compared to those treated with EMVs-free supernatants (EFS) from *S*. *mutans*, underscoring the role of EMVs in modulating protein ubiquitination in *C*. *albicans* (Fig 3B).

### Comprehensive proteomics reveals ubiquitination sites and functions in *C*. *albicans*

**Fig 4:**
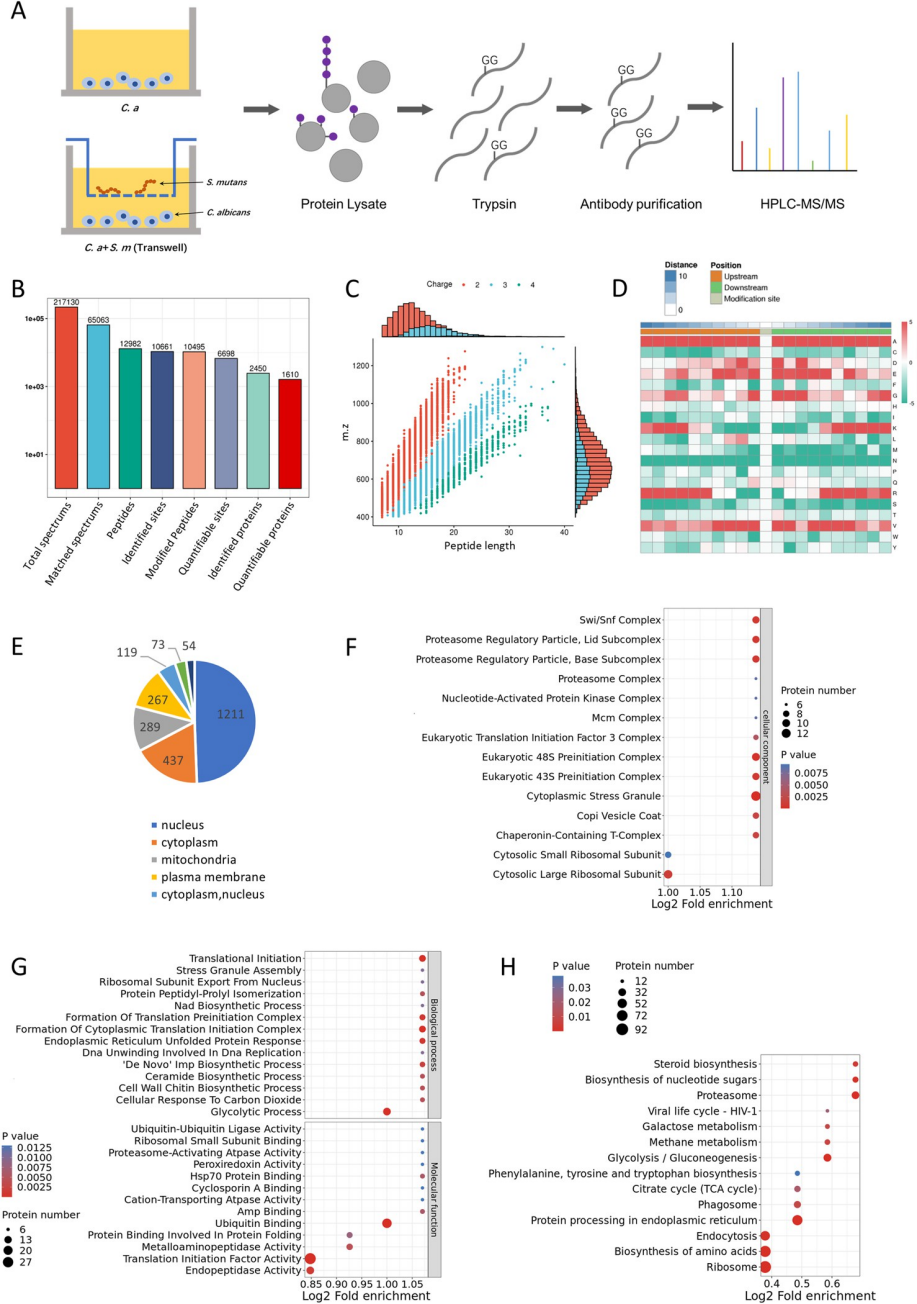
Ubiquitination proteomics analysis of in *C*. *albicans*. (A) Proteomics workflow for identifying and quantifying ubiquitinated proteins in *C*. *albicans*. (B) Statistical overview of ubiquitinated protein identification in *C*. *albicans*. (C-H) Various analyses depicting the ubiquitination landscape: peptide length distribution, motif analysis, subcellular localization, GO enrichment for cellular components, molecular functions, biological processes, and KEGG pathway enrichment.

To identify ubiquitination modification sites on *C*. *albicans* proteins, we deployed quantitative proteomics, enriched for ubiquitinated peptides, to map the ubiquitination sites across the *C*. *albicans* proteome. The process involved protein extraction, digestion, peptide segment enrichment, and identification via HPLC-MS/MS, culminating in the identification of 10,495 ubiquitination modification segments across 2,450 proteins (Fig 4A–4C). Motif analysis indicated a prevalence of hydrophobic residues at ubiquitination sites (Fig 4D). Functional annotation revealed that ubiquitinated proteins are predominantly localized in the nucleus, cytoplasm, mitochondria, and cell membrane, implicating them in critical cellular functions (Fig 4E). GO and KEGG analyses further delineated the biological processes, molecular functions, and metabolic pathways ubiquitinated proteins are involved in, highlighting their role in cellular stress response, translation, protein degradation, chromatin remodeling, and metabolic processes (Fig 4F–4H).

### *S*. *mutans* Induces Widespread shifts in *C*. *albicans* ubiquitination profile

**Fig 5:**
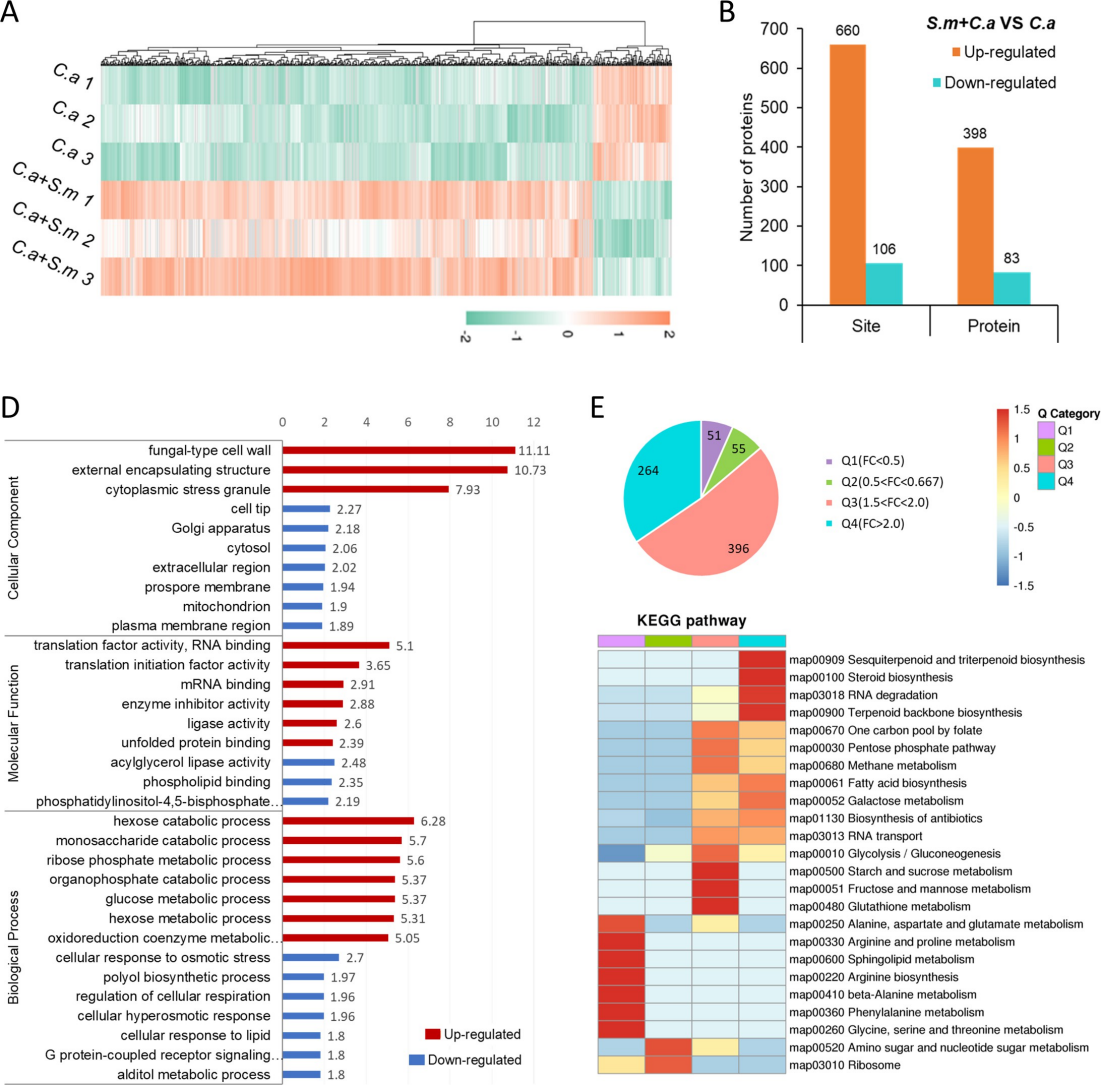
Differential Ubiquitination in *C*. *albicans* under *S*. *mutans* Co-Culture. (A) Heatmap of differential ubiquitination modification sites. (B) Statistics of differential ubiquitination modifications and proteins. (C) GO enrichment analysis of proteins with altered ubiquitination. (D) Cluster and pathway analysis of proteins with differentially modified ubiquitination sites.

We analyzed ubiquitination proteomics in *C*. *albicans* under monoculture condition (*C*.*a* group) and co-culture with *S*. *mutans* in transwell plates (*C*.*a* +*S*.*m* group). Quantitative analysis of ubiquitination in *C*. *albicans* revealed extensive shifts in the ubiquitination landscape upon co-cultivation with *S*. *mutans*. We noted significant upregulation in ubiquitination across many proteins and modification sites, with a heatmap and statistical analysis illustrating these changes (Fig 5A and 5B). Specifically, 398 proteins displayed upregulated ubiquitination modifications, with 660 corresponding upregulated modification sites. 83 proteins exhibited downregulated ubiquitination modifications, accompanied by 106 downregulated modification sites. These findings collectively highlight substantial alterations in the ubiquitination modification landscape of *C*. *albicans* when co-cultured with *S*. *mutans*.

Functional analysis of differentially ubiquitinated proteins highlighted their involvement in various cellular processes and localizations, indicating a broad impact of *S*. *mutans* on *C*. *albicans*’ ubiquitination profile (Fig 5C). We found that proteins with upregulated ubiquitination modifications were primarily associated with molecular functions such as translation factor activity, RNA binding, enzyme inhibitor activity, ligase activity, and unfolded protein binding. In terms of biological processes, proteins with upregulated ubiquitination modifications were mainly involved in processes related to hexose, monosaccharide, ribose phosphate, glucose metabolism, and coenzyme metabolism. Proteins with upregulated ubiquitination modifications were predominantly localized to fungal cell walls and cytoplasmic stress granules, while those with downregulated modification levels were mainly found in the Golgi apparatus, mitochondria, and cell membrane regions. Other modified proteins located in membrane and extracellular spaces may be associated with osmoprotection, transport, adhesion, and resistance to environmental changes.

Further cluster analysis and pathway investigation into differentially ubiquitinated proteins underscored the metabolic pathways and physiological processes affected, suggesting a regulatory role of *S*. *mutans* on *C*. *albicans*’ core metabolic pathways through ubiquitination modulation (Fig 5D). Proteins with downregulated ubiquitination modification sites were primarily enriched in various amino acid metabolic pathways, including pathways related to alanine, aspartate, and glutamate metabolism, as well as amino sugar and nucleotide sugar metabolism, and ribosome pathways. In contrast, proteins with upregulated ubiquitination modification sites were predominantly associated with pathways such as antibiotic biosynthesis, glycolysis/gluconeogenesis, fructose and mannose metabolism, pentose phosphate pathway, galactose metabolism, RNA transport, and degradation pathways. Notably, glycolysis/gluconeogenesis-related pathways constitute central energy metabolism pathways within cells. Key enzymes in these pathways, such as fructose-1,6-bisphosphate aldolase, ATP-dependent 6-phosphofructokinase, glucose kinase, and glycerolaldehyde-3-phosphate dehydrogenase, exhibited upregulated ubiquitination modifications. Additionally, differential modification proteins were found to be enriched in the pentose phosphate pathway, which is a major source of NADPH and ribose-5-phosphate. Collectively, these findings suggest that *S*. *mutans* may regulate the core metabolic pathways of *C*. *albicans* by modulating the ubiquitination modification of key enzymes, thereby influencing its growth and adaptability.

### Integrated proteomics implicates ubiquitination in protein turnover

**Fig 6:**
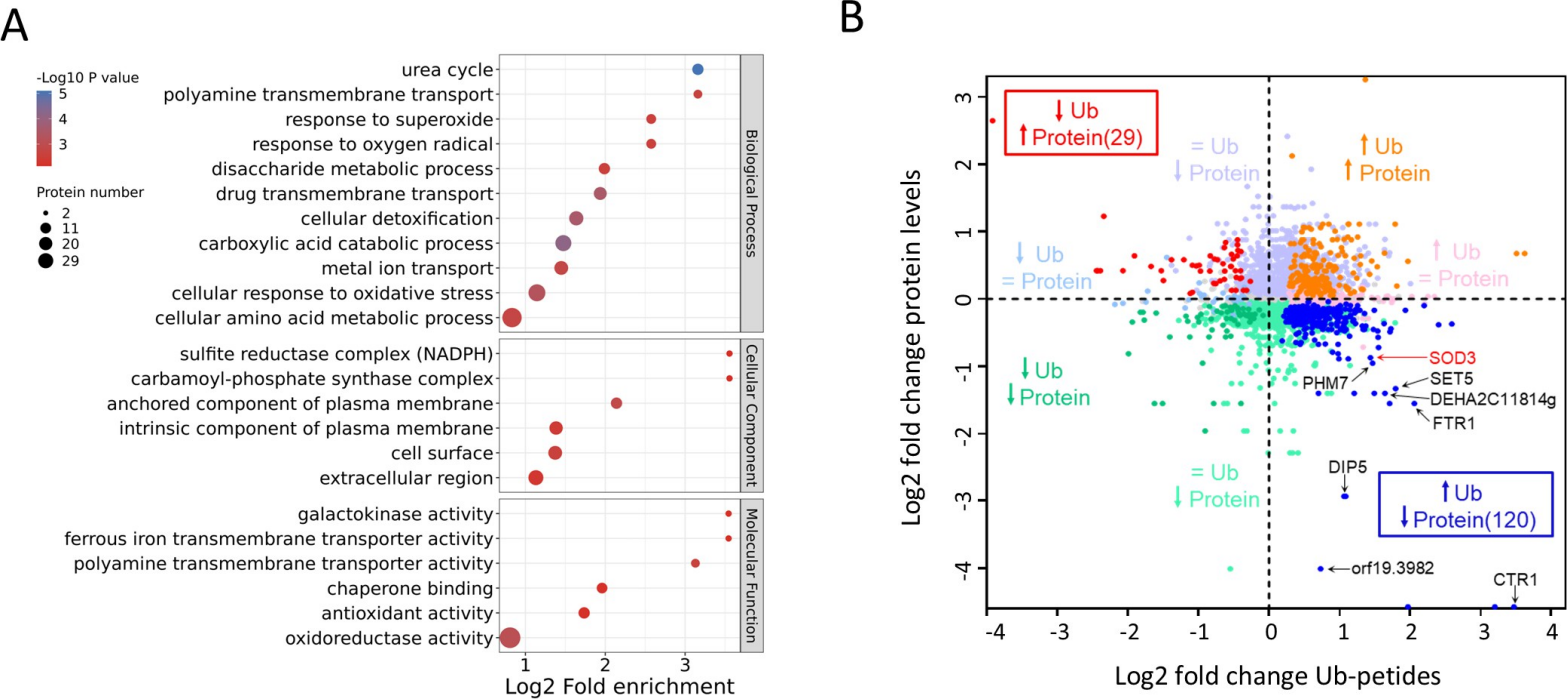
Comprehensive Analysis of Proteomics and Ubiquitination Proteomics in *C*. *albicans*. (A) Enrichment analysis of biological processes, cellular components and molecular functions of differential protein based on GO analysis. (B) Comparison of Ubiquitin (Ub) peptides and corresponding protein levels.

To further elucidate the role of *S*. *mutans* in the physiological process of *C*. *albicans*, we analyzed the proteomes of *C*.*a* group and *C*.*a* +*S*.*m* group. GO analysis of the differentially expressed proteins highlighted a significant enrichment in biological processes related to the cellular response to oxidative stress, superoxide, and oxygen radicals, suggesting a substantial impact of *S*. *mutans* on the redox homeostasis of *C*. *albicans* (Fig 6A). And our proteomic analysis also identified specific changes in the abundance of E3 ligases and deubiquitinases in response to *S*. *mutans*. Notably, the E3 ligase orf19.7445 was downregulated, while RTT101 and orf19.4191.1 were upregulated. Conversely, deubiquitinase orf19.5680 showed downregulation, and DOA4 and orf19.9344 were upregulated. These alterations were consistent with changes in *C*. *albicans* gene expression, yet the expression of the ubiquitin gene ubi4 remained stable, suggesting a selective regulatory effect of *S*. *mutans* on the ubiquitination machinery (S2A and S2B Fig).

By combining proteomics and ubiquitination modification proteomics, we presented the changes in protein abundance and ubiquitination modification of C. albicans caused by S. mutans in Fig 6B. Interestingly, the ubiquitination modifications of certain proteins, such as copper transporter CTR1, iron transporter FTR1, methyltransferase SET5, and superoxide dismutase SOD3, were significantly upregulated under co-culture conditions. This upregulation in ubiquitination corresponded with a marked decrease in their protein levels, indicating that *S*. *mutans* may induce a regulatory shift promoting ubiquitination-related protein degradation. The altered ubiquitination and consequent degradation of SOD3 are particularly noteworthy, as this enzyme plays a crucial role in managing reactive oxygen species (ROS) levels and cell signaling. The ubiquitination-induced modulation of SOD3 and other proteins suggests a complex interplay where *S*. *mutans* influences *C*. *albicans*’ response to oxidative stress and potentially its pathogenicity and survival strategies (Fig 6B).

### *S*. *mutans* modulates Ubiquitination-Dependent cellular functions in *C*. *albicans*

**Fig 7:**
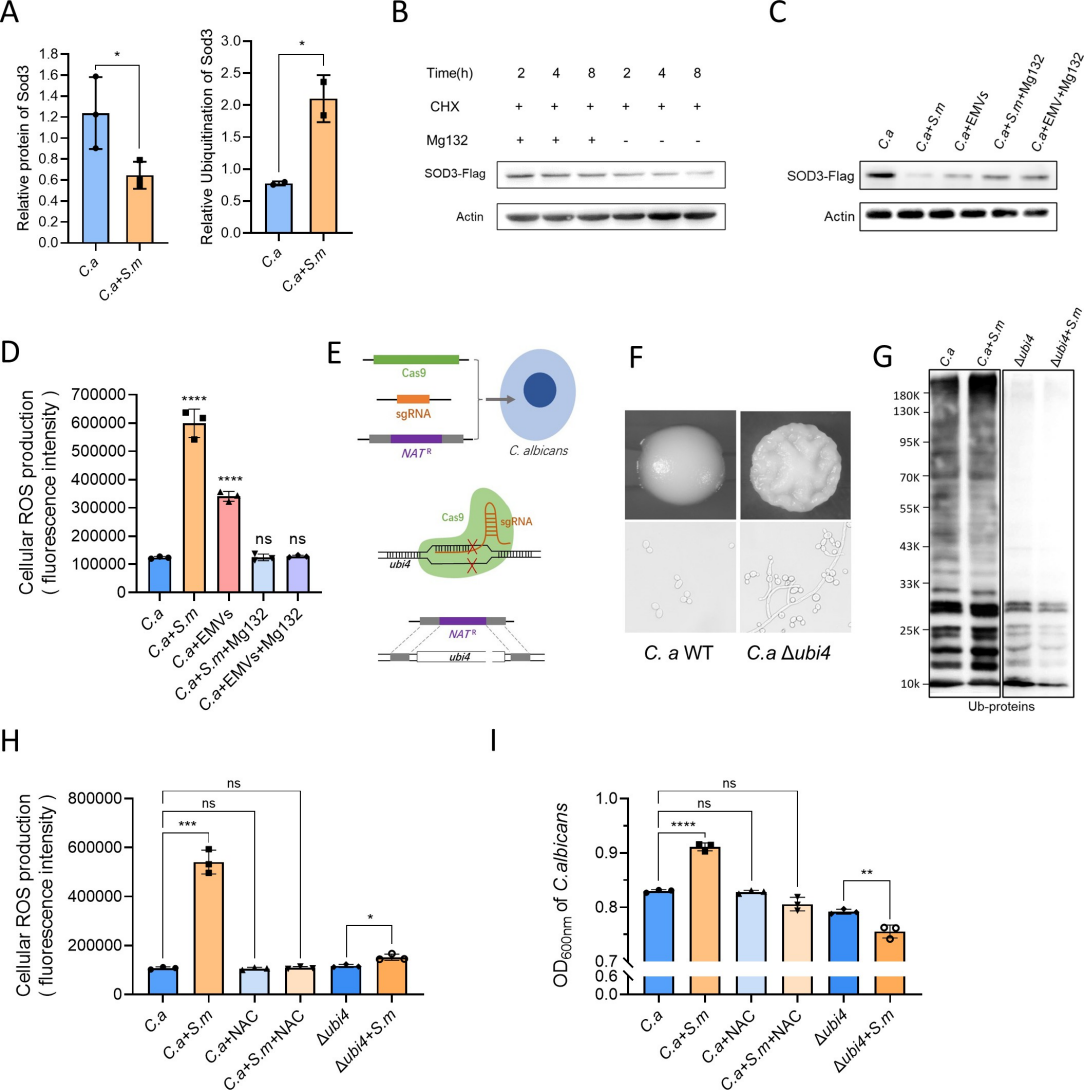
*S*. *mutans* Mediates SOD3 Ubiquitination-Induced Degradation, Elevating Intracellular ROS Levels, and Promoting *C*. *albicans* Growth. (A) Proteomics of *C*. *albicans* SOD3 ubiquitination. (B) MG132 inhibition of SOD3 degradation. (C) Western blot detection of SOD3 protein levels. (D) Intracellular ROS detection in *C*. *albicans*. (E) CRISPR/Cas9 construction of Δubi4 strain. (F) Morphological changes in Δubi4 strain. (G) Immunoblot of protein ubiquitination modifications. (H) ROS levels in *C*. *albicans* under *S*. *mutans* influence. (I) Impact of *S*. *mutans* on *C*. *albicans* growth and ROS modulation. *S*.*m*: *Streptococcus mutans*, *C*. *a*: *Candida albicans*, EMV: *Streptococcus mutans* extracellular membrane vesicles, NAC: N-Acetyl-L-cysteine antioxidant, *: P < 0.05, **: P < 0.01, ns: no statistically significant difference.

Our proteomic analysis highlighted a significant upregulation in the ubiquitination of *C*. *albicans* superoxide dismutase (SOD3), showing a 2.707-fold increase, while its protein content decreased to 0.545 of its original level (Fig 7A). Crispr transient expression technology was employed to add a Flag tag to the C-terminus of SOD3 protein in *C*. *albicans* SC5314. Cycloheximide (CHX) was used to inhibit protein biosynthesis in *C*. *albicans*. Western blot results demonstrated that the proteasome inhibitor MG132 could block the degradation of SOD3, indicating that SOD3’s ubiquitination modification mediates proteasomal degradation (Fig 7B). When co-cultured with *S*. *mutans* and *S*. *m* EMVs, we observed a decrease in SOD3 protein levels in *C*. *albicans*, alongside an increase in reactive oxygen species (ROS) as detected by the DCFH-DA fluorescence probe. Notably, the presence of MG132 mitigated the degradation of SOD3 and consequently suppressed the rise in intracellular ROS levels (Fig 7C and 7D). These findings suggest that *S*. *mutans*, possibly through its EMVs, enhances the ubiquitination and subsequent proteasomal degradation of SOD3, leading to an increase in ROS within *C*. *albicans*.

**Fig 8:**
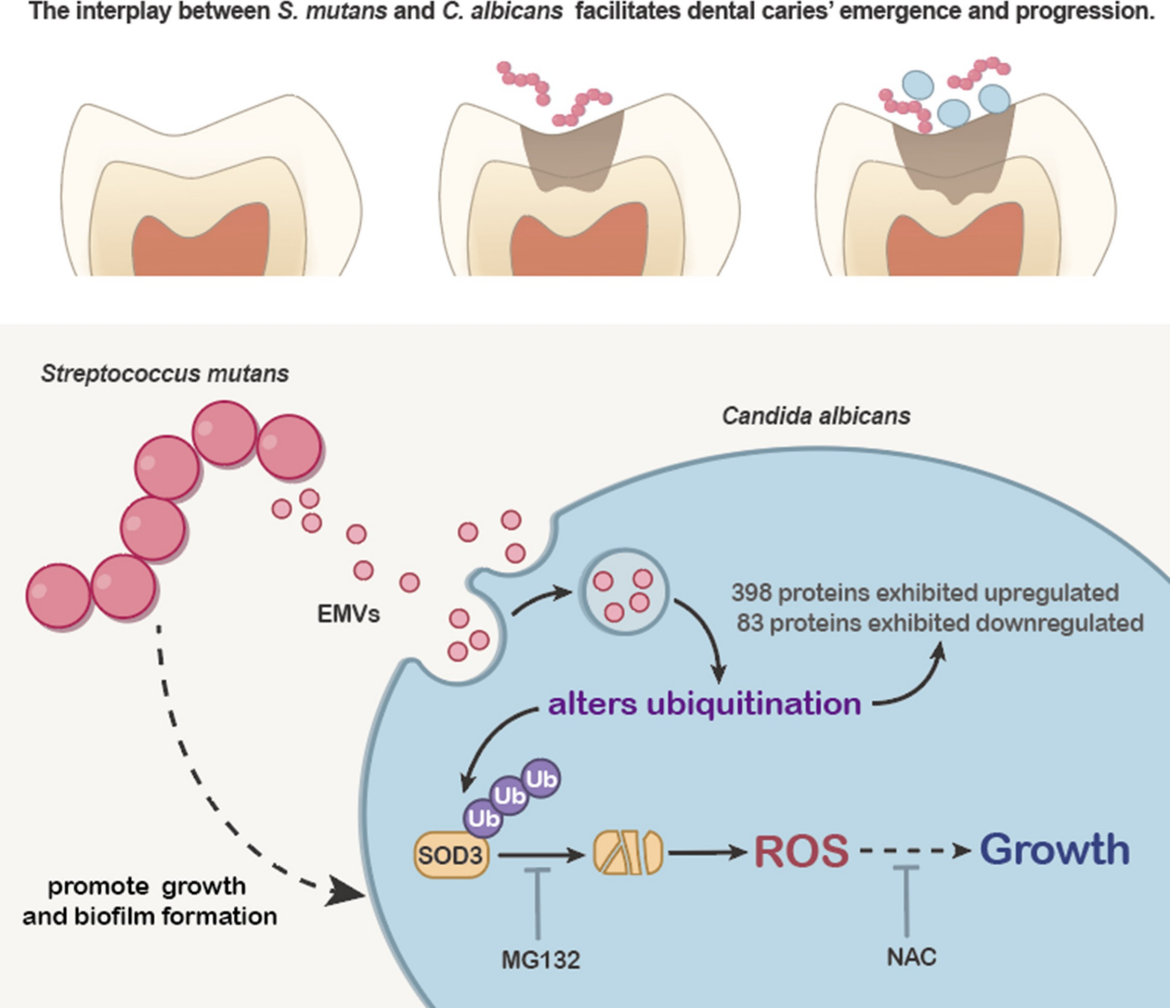
Schematic showing a proposed model for the role of *S*. *mutans* in modulating ubiquitination of SOD3, influencing ROS levels, and potentially impacting growth of *C*. *albicans*.

In order to further elucidate the role of ubiquitination modification regulation of *C*. *albicans* by *S*. *mutans*, Crispr transient expression technology was employed to create a polyubiquitin gene *ubi4* knockout strain of *C*. *albicans* (Fig 7E). At 37°C, the wild-type *C*. *albicans* exhibited typical budding yeast morphology, while the SC5314 Δubi4 strain showed an aberrant mix of pseudohyphae and hyphal cells, indicating a disruption in normal cell morphology due to the lack of functional ubi4 (Fig 7F). In the wild-type, co-culture with *S*. *mutans* altered the ubiquitination profile of certain proteins. However, in the Δubi4 strain, a marked reduction in ubiquitination was observed, highlighting the pivotal role of polyubiquitin in ubiquitination processes within *C*. *albicans* (Fig 7G). When exposed to *S*. *mutans*, the wild-type *C*. *albicans* showed a significant increase in intracellular ROS levels. In contrast, the Δubi4 strain did not exhibit the same increase in ROS, suggesting a direct correlation between ubiquitination and ROS production. Furthermore, treatment with N-acetyl-L-cysteine (NAC), an antioxidant, not only reduced ROS levels but also attenuated the growth-promoting effects of *S*. *mutans* on *C*. *albicans* (Fig 7H and 7I). These results collectively underscore the newly identified role of *S*. *mutans* in modulating ubiquitination, influencing ROS levels, and concomitantly promoting the growth of *C*. *albicans* (Fig 8). These processes likely involve a complex interplay of incompletely understood cellular mechanisms that should be explored further in future studies.

## Discussion

The "contact-independent" interaction between *S*. *mutans* and *C*. *albicans* is likely facilitated by extracellular membrane vesicles (EMVs). *S*. *m* EMVs contain proteins, extracellular DNA, and other biologically active substances [22]. Previous studies have demonstrated that *S*. *m* EMVs are implicated in cell wall synthesis, bacterial adhesion, biofilm formation, and can promote biofilm formation of various microorganisms in the oral cavity, including *Streptococcus mitis*, *Streptococcus oralis*, and *C*. *albicans* [15,16,23]. *S*. *m* EMVs enrich the extracellular matrix of *C*. *albicans* biofilms and promote carbohydrate metabolism, thus enhancing biofilm formation [17,24]. Similar to previous studies, we found that *S*. *m* EMVs can promote the growth and biofilm formation of *C*. *albicans*. Specifically, through CLSM, we observed the co-localization of *S*. *m* EMVs with *C*. *albicans*, suggesting that might underpin these effects.

Our ubiquitination proteomics revealed that approximately 49% of ubiquitinated proteins in *C*. *albicans* localize within the cell nucleus, implicating ubiquitination in critical nuclear processes such as DNA replication, gene expression, damage repair, and recombination. This suggests that nuclear protein ubiquitination in *C*. *albicans* plays a role in regulating and maintaining cellular biological processes, potentially affecting physiological pathways related to biosynthesis, energy metabolism, and substance interactions. These insights provide a valuable understanding of the molecular functions and physiological regulation of ubiquitination modification in *C*. *albicans*.

Prior research has shown that *C*. *albicans* cannot effectively metabolize sucrose, whereas *S*. *mutans* GtfB can break down sucrose into reducing sugars that *C*. *albicans* can utilize, indirectly promoting its growth and metabolism [25]. In examining the effect of *S*. *mutans* on ubiquitination in *C*. *albicans*, our proteomic analysis showed significant changes in the ubiquitination of key enzymes involved in crucial metabolic pathways. This suggests that *S*. *mutans* might regulate the ubiquitination of key enzymes in sugar metabolism of *C*. *albicans*, influencing enzyme activity or substrate binding, thus directly affecting energy metabolism. These findings provide a new perspective and theoretical foundation for further exploration of the interaction mechanisms between *S*. *mutans* and *C*. *albicans*.

Our study also highlighted the role of reactive oxygen species (ROS) in the interaction between *S*. *mutans* and *C*. *albicans*. Excessive ROS is widely recognized as a harmful reactive substance that can damage cellular proteins, lipids, and nucleic acids, leading to oxidative stress [26]. Overproduction of ROS in Candida albicans has been shown to induce apoptosis [27]. Although ROS has the potential for cellular toxicity, at low concentrations, ROS plays a crucial role in normal physiological and pathological processes, contributing to pathogen resistance and cell signaling [28,29]. Evidence suggests that the generation of ROS, such as O_2_- or H_2_O_2_, is an integral part of the pathogen defense mechanism [30,31]. Moderate ROS levels can promote tumor cell proliferation and serve as second messengers in growth factor activation through the PI3K/AKT/mTOR and MAPK/ERK signaling cascades [32,33]. The proliferation and cell viability of microglial cells have been linked to ROS generated through PI3Kγ [34]. Cardiac fibroblast proliferation is associated with higher ROS levels [35]. While excessive ROS can be detrimental, causing oxidative stress and apoptosis, moderate levels are crucial in normal physiological and pathological processes. We found that co-culture with *S*. *mutans* promotes an increase in intracellular ROS levels in *C*. *albicans*, enhancing its growth and biofilm formation. The application of NAC effectively counteracted these effects by inhibiting intracellular ROS production, indicating the pivotal role of ROS in the growth of *C*. *albicans* within dual species biofilms.

Superoxide dismutase (SOD), especially SOD3, plays a critical role in catalyzing the conversion of ROS into less harmful molecules [36,37]. SOD activity governs the levels of various ROS and reactive nitrogen species, limiting the potential toxicity of these molecules while also regulating cellular signal transduction. Given the diverse sources and slow diffusion of ROS, aerobic organisms possess various SOD proteins localized to different subcellular compartments to finely control cellular ROS homeostasis [38,39]. *C*. *albicans* possesses six SOD enzymes, including Cu/Zn-containing SOD1 in the mitochondrial matrix, Mn-containing SOD2 in the cytoplasm, Cu-containing SOD4, SOD5, and SOD6 in the extracellular space, and the predicted Mn-containing SOD3 presumably located in the cytoplasm [40–43]. Previous studies have indicated that SOD3 expression is induced in *C*. *albicans* cells under copper deficiency conditions [44]. While SOD3 is predicted as a cytoplasmic protein, it remains unclear whether it can enter the mitochondrial intermembrane space to regulate ROS release. Our experiments indicate that *S*.*m* EMVs can increase the ubiquitination and subsequent degradation of *C*. *albicans* SOD3, leading to elevated intracellular ROS levels, and concomitantly promoting fungal growth. Given the intricate nature of ROS regulation and the diverse roles of SOD isoforms, further research is needed to understand the subcellular localization of these enzymes and the specific mechanisms through which *S*. *mutans* influences *C*. *albicans* physiology and pathogenicity.

In conclusion, this study has uncovered significant aspects of the interaction between *S*. *mutans* and *C*. *albicans*, particularly the role of EMVs, ubiquitination, and ROS in mediating their complex interplay. The implications of these findings extend to our understanding of oral biofilm dynamics, pathogenicity, and potential therapeutic strategies targeting microbial interactions within the oral microbiome. Further research in these areas is essential to fully elucidate the mechanisms at play and harness this knowledge for clinical applications.

## Materials and methods

### Ethics statement

The experiments involving rats in this study were performed in accordance with the protocols and procedures approved by the institutional Animal Care and Use Committee of West China Hospital of Stomatology, Sichuan University (approval number WCHSIRB-D-2023-130). The animal care and use protocol adhered to the Chinese National Laboratory Animal-Guidelines for Ethical Review of Animal Welfare.

### Bacterial strains and growth conditions

*S*. *mutans* UA159 (ATCC 700610) and *C*. *albicans* SC5314 (ATCC MYA-2876), used in this study, were obtained from the Oral Microbiome Bank of China [45]. *C*. *albicans* SC5314 Δ*ubi4* and SC5314 SOD3-Flag were constructed using the CRISPR-Cas9 transient expression method as previously described [46]. Primer sequences used in gene editing are listed in S1 Table. All resulting gene mutations were verified by DNA sequencing. These strains were typically cultured at 37°C under aerobic conditions (5% CO2, 95% air) in ultra-filtered tryptone yeast extract medium (UFTYE) (pH = 7.0 for *S*. *mutans* and pH = 5.5 for *C*. *albicans*) [7,8,25]. For co-culture experiments, an overnight culture of *S*. *mutans* UA159 was adjusted to OD600nm of ∼ 0.3, *C*. *albicans* SC5314 was adjusted to OD600nm of ∼ 0.375. 100ul of *C*. *albicans* and 800ul of UFTYE medium containing 1% (wt/vol) sucrose and 1% (wt/vol) glucose were added to the plate bottom of a 24-well transwell plates with a 0.4 μm pore size PE membrane (Corning, NY, United States). In the apical compartment, 100ul of *S*. *mutans* or *S*.*m* EMVs were added according to experimental groups.

### Growth characteristics of *C*. *albicans*

Growth kinetics: *C*. *albicans* and *S*. *mutans* were co-inoculated in transwell plates using the above-mentioned culture conditions. The plate was placed in a 5% CO2 incubator at 37°C. Every 2 hours, the suspension of *C*. *albicans* was transferred to a new 96-well plate. This experiment was performed with three biological replicates and three technical replicates.

Colony forming units (CFU): Following the above culture conditions for 24 hours, the suspension of *C*. *albicans* was diluted to different concentrations. The prepared suspensions were plated on YPD solid medium and incubated at 37°C for 24 hours.

Biofilms: Biofilms of *C*. *albicans* were formed in transwell plates using UFTYE medium containing 15% fetal bovine serum (Gibco Laboratories, Gaithersburg, MD, United States), 1% (wt/vol) sucrose, and 1% (wt/vol) glucose for 24 hours. The biofilms were washed three times with sterile PBS to remove supernatant and planktonic cells. Methanol was added for fixation, followed by 0.1% (wt/vol) crystal violet staining for 5 minutes. After three additional washes with sterile PBS, crystal violet was extracted with 95% ethanol at 37°C for 30 minutes. Then, 100 μL of the ethanol eluate was transferred to a new 96-well plate and detected at 570 nm [47]. This experiment was conducted with three biological replicates and three technical replicates.

### Preparation and characterization of *S*.*m* EMVs

EMVs Extraction: *S*. *mutans* were cultured in 500 mL of BHI medium at 37°C for 16 hours. The culture was centrifuged (6000×g, 15 min, 4°C) to remove bacteria. The supernatant was further centrifuged (10000×g, 15 min, 4°C) to eliminate bacterial debris. The resulting supernatant was filtered through a 0.22 μm membrane (Millipore, MMAS, United States), followed by ultracentrifugation (100000×g, 70 min, 4°C). After a single wash with sterile PBS, the EMVs were resuspended in 1 mL of sterile PBS.

Morphological Analysis: Ten microliters of EMVs suspension were adhered to formvar/carbon-coated nickel TEM grids, negatively stained with 3% uranyl acetate for 1 minute, rinsed with ddH2O, and then observed and identified for the presence of EMVs using transmission electron microscopy (H7650, Hitachi, Japan) at 80 kV acceleration voltage.

Nanoparticle Tracking Analysis (NTA): NTA was performed using a NanoSight NS300 equipped with a 488 nm laser and an automatic injection pump [48]. Recorded videos were processed using NTA 3.2 software. *S*.*m* EMVs, diluted 1:100 in PBS, were loaded using the automatic injection pump. The initial speed setting was fixed at 1000 for sample loading and chamber filling, which was subsequently reduced to 25 for video recording. Videos were captured for 60 seconds with a camera level setting of 14, and a detection threshold of 3 was applied.

### Labeling and tracing of *S*.*m* EMVs

DID (Thermo Fisher Scientific, Pittsburgh, PA, United States) was added to the *S*.*m* EMVs PBS solution, and the mixture was incubated at room temperature in the dark for 30 minutes. Then, 1% BSA was added to quench excess dye. The mixture was subjected to ultracentrifugation (100000×g, 70 min, 4°C), followed by a single wash with sterile PBS. The labeled *S*.*m* EMVs were resuspended in sterile PBS for further use. These labeled *S*.*m* EMVs were co-incubated with approximately 1×10^6^ CFU/mL of *C*. *albicans* yeast cells for 1 hour. After incubation, the medium was removed, and the samples were washed twice with physiological saline. *C*. *albicans* was stained by adding 50 μL of 5 μM SYTO 9 (Invitrogen Corp., Carlsbad, CA, United States) to the confocal dish samples, followed by incubation at room temperature in the dark for 15 minutes and subsequent washing with physiological saline three times. A drop of anti-fade fluorescence quencher was added, and the samples were either stored at -20°C in the dark or immediately observed and imaged using an Olympus FLUPVIEW FV3000 confocal laser scanning microscope (Olympus Corp., Tokyo, Japan). Finally, three-dimensional reconstruction of the biofilm was performed using Imaris 7.0.0 (Bitplane, Zürich, Switzerland).

### Western Blot (WB)

After samples harvesting and lysis, crude extracts were prepared and protein levels were assessed using Western blotting. Cell lysates were separated by SDS-PAGE (15% gel for SOD3-Flag detection, 11% gel for ubiquitination analysis) and transferred onto NC membranes. Membranes were blocked with 5% skim milk and incubated overnight at 4°C with either Anti-Ubiquitin Rabbit mAb (NT) (Hangzhou Jingjie Biotechnology Co) or antibody against FLAG (Agilent). Subsequently, secondary antibodies were applied and incubated at room temperature for 1 hour, followed by image acquisition using a gel imaging system. For SOD3 degradation assessment, cells were treated with 10 μM CHX (Cell Signaling Technology) to block protein synthesis for 24 hours prior to sample collection, and 20 μM MG132 (Cell Signaling Technology) was added to the cells 2 hours, 4 hours, and 8 hours before harvest.

### Protein extraction

For protein extraction, *C*. *albicans* samples, each with three biological replicates, were collected. Four volumes of SDS lysis buffer (containing 1% protease inhibitor, 10 mM dithiothreitol, and 50 μM PR-619) were added to each sample, followed by sonication. Protein concentrations were determined using the BCA assay after protein extraction with Tris-equilibrated phenol, 0.1 M ammonium acetate/methanol, and 8 M urea.

### Enzymatic digestion

Equal amounts of protein from each sample were enzymatically digested. After precipitation with pre-chilled acetone, the pellets were resuspended in 200 mM TEAB and digested overnight with trypsin. Reduction was performed with 5 mM dithiothreitol (DTT) at 56°C for 30 minutes, followed by alkylation with 11 mM iodoacetamide (IAA) at room temperature in the dark for 15 minutes.

### Enrichment for modified peptides

Peptide segments were dissolved in IP buffer (containing 1 mM EDTA, 100 mM NaCl, 0.5% NP-40IP, and 50 mM Tris-HCl, pH 8.0). The supernatant was transferred to pre-washed ubiquitin resin (PTM-1104, Hangzhou Jingjie Biotechnology Co., Ltd), shaken overnight at 4°C, and subsequently washed four times with IP buffer, followed by two washes with deionized water. Finally, bound peptide segments were eluted, vacuum freeze-dried, and desalted before liquid chromatography-mass spectrometry (LC-MS) analysis. This step was omitted in the proteomics analysis.

### Peptides analysis by high-performance liquid chromatography-tandem mass spectrometry (HPLC-MS/MS)

LC-MS Analysis was carried out using an Easy-nLC1000 nano-flow liquid chromatography system (Thermo Fisher Scientific) coupled with a quadrupole-orbitrap hybrid mass spectrometer (Q Exactive Plus, Thermo Fisher Scientific). Peptides were dissolved in solvent A (0.1% formic acid) and directly loaded onto a custom-made reversed-phase analytical column (length 15 cm, inner diameter 75 μm). The gradient included solvent B, 0–40 min, increasing from 7% to 24%; 40–52 min, increasing from 24% to 32%; 52–56 min, increasing from 32% to 80%; and 56–60 min, maintaining at 80%, with a flow rate of 450 nL/min. For proteomics LC gradient settings: 0–70 min, gradient from 6% to 24% B; 70–82 min, gradient from 24% to 35% B; 82–86 min, gradient from 32% to 80% B; and 86–90 min, maintained at 80%, with a flow rate of 450 nL/min. Peptides were ionized in the capillary ion source and then analyzed in the timsTOF Pro 2 mass spectrometer. Ion source voltage was set at 1.65 kV. High-resolution TOF detection and analysis of peptide parent ions and their secondary fragments were performed. The secondary mass spectrometry scan range was set to 100–1700. Data were acquired in parallel accumulation-serial fragmentation (PASEF) mode. For each primary spectrum acquisition, 10 PASEF mode scans were performed for secondary ion charge states in the range of 0–5, with a dynamic exclusion time set to 30 seconds.

### Mass spectrometry raw data search

For the database search of secondary mass spectrometry data, MaxQuant software (v1.6.15.0) was used, and the reference database was Blast_Candida_albicans_strain_SC5314_ 237561_PR_20220418.fasta (6035 sequences). Both main and first searches had a primary parent ion mass tolerance of 20 ppm and a secondary fragment ion mass tolerance of 20 ppm. Cysteine alkylation was set as a fixed modification, while variable modifications included methionine oxidation, N-terminal acetylation, and ubiquitination of lysine residues. False discovery rates (FDRs) for peptide-spectrum match and protein identification were set at 1%.

Functional Annotation: Gene Ontology (GO) annotation of the proteome was performed using the UniProt-GOA database (http://www.ebi.ac.uk/GOA/). InterProScan was used for functional description annotation based on protein sequences and the InterPro structure domain database (http://www.ebi.ac.uk/interpro/). The Kyoto Encyclopedia of Genes and Genomes (KEGG) database (https://www.kegg.jp/kegg/) was employed for pathway annotation. Subcellular localization prediction was conducted using the CELLO subcellular localization prediction software for prokaryotes. Homologous clustering and classification of modified proteins were performed based on the Clusters of Orthologous Groups (COG) database (http://www.ncbi.nlm.nih.gov/COG).

#### Functional enrichment

Biological processes, cellular components, and molecular functions were classified according to GO annotation. KEGG database was used to identify enriched pathways, and InterPro database was used for enriching protein structural domains. For all enrichment methods mentioned above, a two-tailed Fisher’s exact test was used to test the significance of differential expression proteins among all identified proteins. When the adjusted P-value was less than 0.05, differences were considered significant.

#### Function-Based clustering

After function enrichment, all categories and their P-values were compiled. We filtered categories that were significantly enriched (P < 0.05) in at least one cluster, and then transformed these P-values into matrices with the formula X = -log10 (P-value). Subsequently, Z-scores were calculated for each functional category. These Z-scores were clustered hierarchically using one-way clustering (Euclidean distance, average linkage) in Genesis. Visualization of cluster membership was achieved using the heatmap.2 function from the gplots R package.

### Analysis of *C*. *albicans* gene expression by real-time PCR

Total RNA extraction from cell pellets was carried out using RNAiso Plus (Takara Bio Inc., Otsu, Japan) following the manufacturer’s instructions. The RNA purity and concentration (A260/A280) were determined using a NanoDrop 2000 spectrophotometer (Thermo Fisher Scientific, Pittsburgh, PA, USA). To perform reverse transcription of RNA, we used a PrimeScript RT reagent kit with gDNA Eraser (Takara Bio Inc., Otsu, Japan) following the manufacturer’s protocol. The primer sequences for real-time PCR are provided in S2 Table. Amplification and quantification of target RNA were performed using SYBR Premix Ex Taq II (2×) (Takara Bio Inc., Otsu, Japan) on a LightCycler 480 Real-Time System. Gene expression fold changes were calculated using the 2^–ΔΔCt^ method. This experiment was conducted with three biological replicates and three technical replicates.

#### Reactive Oxygen Species (ROS) assay

Intracellular ROS levels in *C*. *albicans* were measured using a ROS assay kit with the DCFH-DA probe (Beyotime Biotechnology, JiangSu, China). DCFH-DA was diluted in culture medium at a 1:1000 ratio to achieve a final concentration of 10 μM/L. Harvested *C*. *albicans* were washed and resuspended in PBS. The yeast cell suspension was adjusted to OD600 nm ∼ 0.375 (approximately 1×10^7^ CFU/ml). After centrifugation, the cells were resuspended in the diluted DCFH-DA and incubated at 37°C for 20 minutes. The suspension was gently inverted every 3–5 minutes to ensure thorough contact between the probe and the cells. After incubation, cells were washed three times with PBS to remove excess DCFH-DA that had not entered the cells. Subsequently, the cells were collected and fluorescence intensity was measured in a 96-well plate using a fluorescence microplate reader with excitation at 488 nm and emission at 525 nm. This experiment was performed with three biological replicates and three technical replicates.

#### Dental caries rat model

Specific pathogen-free Sprague Dawley (SD) rats (Chengdu Dossy Experimental Animals Co., Ltd, China) at the age of 3 weeks were obtained and randomly divided into three groups, each containing 7 animals. All rats were given ampicillin (1g/kg) in the drinking water for the first three days, and then the antibiotics were washed out with normal sterile water. Then, *S*. *mutans* or *C*. *albicans* solution (10^8^ CFU/mL, 0.2 mL) were inoculated twice a day for 7 consecutive days. Meanwhile, sucrose-containing water (5% concentration) and cariogenic diet 2000 (TrophicDiet, Trophic Animal Feed, Suzhou, China) were provided [49]. The rats were sacrificed three weeks later, and the maxillary and mandibular molars were obtained. All molars were stained with violet ammonia (0.4% concentration) for 6 hours and then semi-sectioned. Then, a stereomicroscope (Leica EZ4HD; Leica Microsystems AG, Heerbrugg, Switzerland) was used and scored using the Keyes scoring method [50].

### Statistical analysis

Statistical analyses were performed using GraphPad Prism version 8.0 unless otherwise noted. P < 0.05 was considered significant.

## Supporting information

S1 Table The primer sequences for gene editing. (XLSX)

S2 Table The primer sequences for real-time PCR. (XLSX)

S1 Fig *S. mutans* Regulates Protein Ubiquitination in *C. albicans*. On the left is Coomassie Brilliant Blue staining of *C. albicans* total proteins. On the right is immunoblotting of ubiquitination modifications in *C. albicans* total proteins. *S.m: Streptococcus mutans, C.a: Candida albicans*.
(TIF)

S2 Fig Analysis of differential proteomics in *C. albicans*. (A) Protein expression levels of E3 ligases and deubiquitinases from proteomics results. (B) qRT-PCR quantification of E3 ligases, deubiquitinases and UBI4 gene mRNA levels. (C) Enrichment GO analysis of differential protein associated with oxidation reduction. *S.m: Streptococcus mutans, C.a: Candida albicans*, *: P < 0.05, **: P < 0.01, ****: P < 0.0001, ns: no statistically significant difference.
(TIF)
