# Supplementary figures and images for: Streptococcus mutans regulates ubiquitin modification of Candida albicans in the bacterial-fungal interaction
Source: PLoS Pathog. 2025 Feb 3;21(2):e1012887. doi: 10.1371/journal.ppat.1012887 (PMC11838896; doi:10.1371/journal.ppat.1012887)

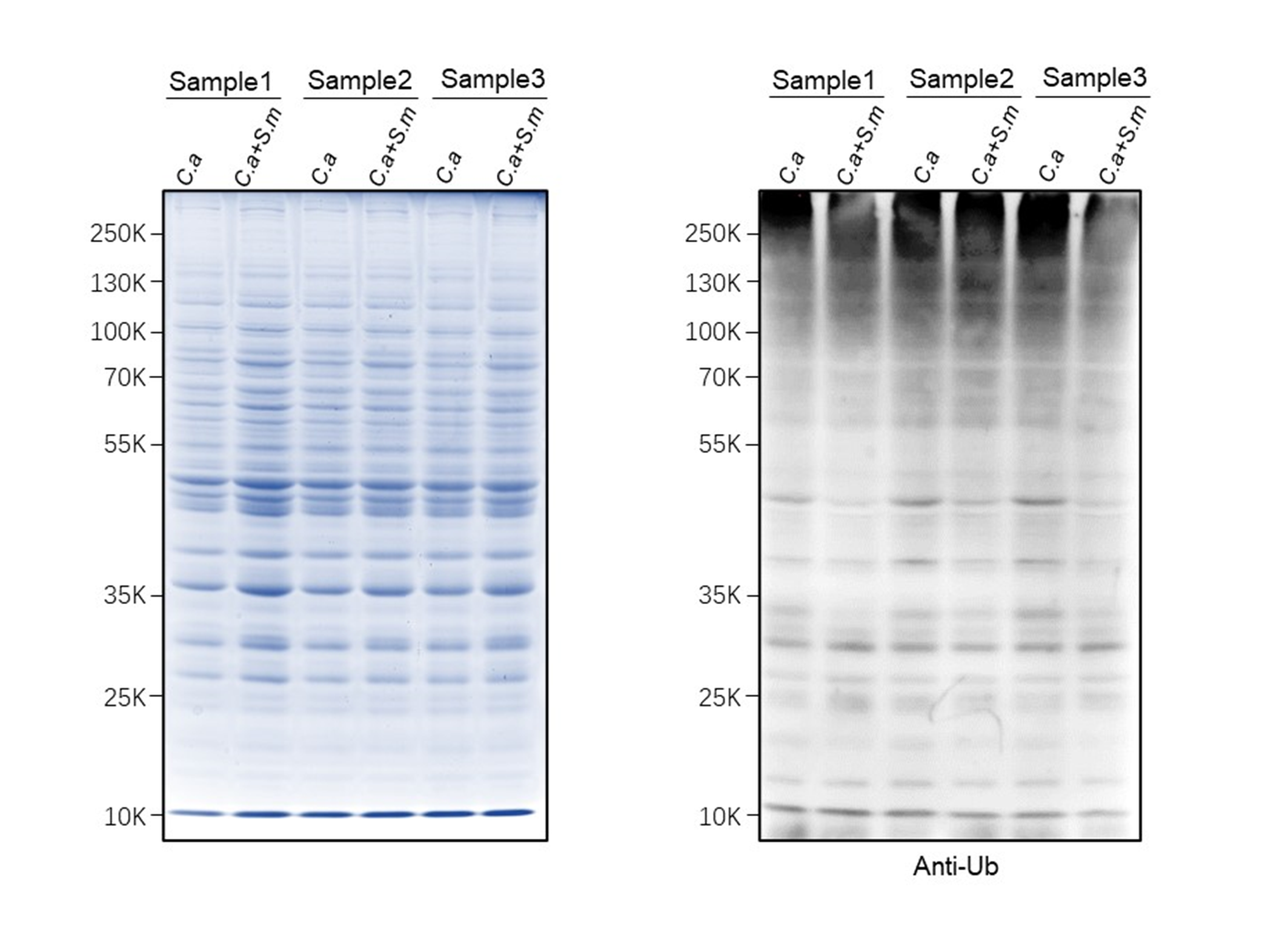

Supplement: S1 Fig — On the left is Coomassie Brilliant Blue staining of C. albicans total proteins. On the right is immunoblotting of ubiquitination modifications in C. albicans total proteins. S.m: Streptococcus mutans, C.a: Candida albicans. (TIF) [file ppat.1012887.s003.tif]

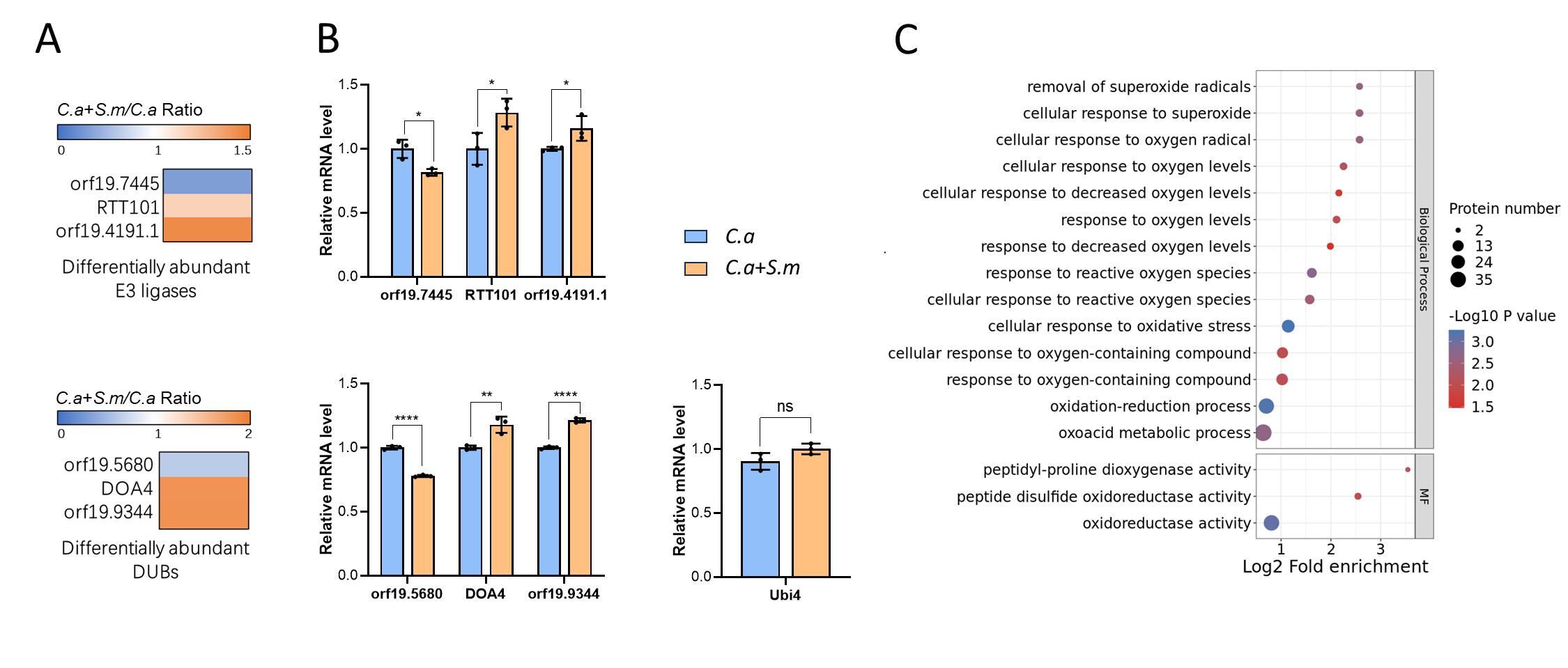

Supplement: S2 Fig — (A) Protein expression levels of E3 ligases and deubiquitinases from proteomics results. (B) qRT-PCR quantification of E3 ligases, deubiquitinases and UBI4 gene mRNA levels. (C) Enrichment GO analysis of differential protein associated with oxidation reduction. S.m: Streptococcus mutans, C.a: Candida albicans, *: P < 0.05, **: P < 0.01, ****: P < 0.0001, ns: no statistically significant difference. (TIF) [file ppat.1012887.s004.tif]
